# Effect of Remineralization Products on the Microtensile Strength of Universal Dentin Bonding Systems

**DOI:** 10.3390/dj13110493

**Published:** 2025-10-24

**Authors:** Andra Claudia Tărăboanță-Gamen, Cristian Marius Toma, Vasilica Toma, Ionuț Tărăboanță, Simona Stoleriu, Veronica Serban Pintiliciuc, Sorin Andrian

**Affiliations:** 1 Faculty of Dental Medicine, Grigore T. Popa University of Medicine and Pharmacy Iasi, Str. Universitatii No. 16, 700115 Iasi, Romania; andra-claudia.gamen@umfiasi.ro (A.C.T.-G.);; 2 Directorate of Programs for Institutional Development—Scientific Research Department, Grigore T. Popa University of Medicine and Pharmacy Iasi, Str. Universitatii No. 16, 700115 Iasi, Romania

**Keywords:** caries-affected dentin, remineralization, CPP-ACP, P11-4 peptide, silver diamine fluoride, universal adhesives, microtensile bond strength

## Abstract

**Background/Objectives**: Adhesion to caries-affected dentin remains challenging due to its altered structure and composition. Remineralizing agents have been proposed to strengthen this substrate and improve bonding. This study evaluated the effect of three remineralization treatments, CPP-ACP, self-assembling peptide P11-4, and silver diamine fluoride (SDF), on the microtensile bond strength (μTBS) of universal adhesive systems applied to caries-affected dentin, using both etch-and-rinse and self-etch strategies. **Methods**: Seventy human molars were sectioned and artificially demineralized to simulate caries-affected dentin. Samples were divided into ten groups: four untreated and six treated with CPP-ACP (MI Paste™), P11-4 (Curodont™ Protect), or SDF (Riva Star™). Universal adhesives were applied via etch-and-rinse or self-etch mode, followed by composite restoration. Microtensile bond strength was measured using a universal testing machine, and results were statistically analyzed with ANOVA and *t*-tests (*p* < 0.05). **Results:** Untreated caries-affected dentin showed significantly lower μTBS than sound dentin (C3: 18.3 ± 5.4 MPa vs. C1: 41.3 ± 2.7 MPa). Remineralization agents improved μTBS considerably. CPP-ACP achieved the highest recovery (S1: 31.8 ± 2.6 MPa; S2: 29.2 ± 4.6 MPa), nearing sound dentin levels. P11-4 yielded moderate gains (S3: 24.4 ± 6.5 MPa; S4: 24.1 ± 4.7 MPa), while SDF provided the lowest, yet significant, improvements (S5: 23.7 ± 7.5 MPa; S6: 21.3 ± 5.3 MPa). Etch-and-rinse generally produced higher μTBS than self-etch, but differences were not statistically significant (*p* > 0.05). **Conclusions**: Pre-treatment of caries-affected dentin with CPP-ACP, P11-4, or SDF enhances universal adhesive bond strength, with CPP-ACP showing the most pronounced effect. Remineralization protocols represent a valuable adjunct in restorative procedures involving compromised dentin.

## 1. Introduction

The application of universal dental adhesives has become a common approach in direct restorative procedures due to their ability to bond reliably with both enamel and dentin using techniques such as etch-and-rinse and self-etch [1]. Contemporary adhesive systems aim to provide durable bonding to various dental substrates, including caries-affected dentin [2]. However, establishing stable adhesion to caries-affected dentin, characterized by partial demineralization while preserving structural integrity, remains a significant clinical challenge [3]. Numerous studies consistently report that the bond strength of composite resins to demineralized dentin is markedly lower than to sound dentin [4,5,6]. This reduction is related to morphological and compositional alterations, including decreased mineral content, exposed and partially denatured collagen, residual organic layers, and bacterial by-products, all of which impair the formation of a uniform hybrid layer [4]. Additionally, caries-affected dentin exhibits increased permeability and heterogeneous demineralization zones, limiting resin monomer penetration and promoting water accumulation at the adhesive interface, thereby increasing susceptibility to hydrolytic and enzymatic degradation [3]. Activation of endogenous enzymes such as matrix metalloproteinases (MMPs) and cysteine cathepsins further accelerates collagen breakdown within the hybrid layer, compromising bond durability [6].

Given the growing emphasis on minimally invasive dentistry, preserving caries-affected dentin has become a priority [6]. Selective removal of only the superficially infected dentin is now preferred, as the remaining affected dentin retains structurally intact collagen fibrils and demonstrates potential for remineralization [4]. Consequently, several in situ remineralization strategies have been introduced to strengthen the dentin matrix and improve long-term adhesive performance [6,7]. Among these, casein phosphopeptide-amorphous calcium phosphate (CPP-ACP), P11-4 self-assembling peptides, and silver diamine fluoride (SDF) have shown promising effects. CPP-ACP (MI Paste™) releases bioavailable calcium and phosphate ions, facilitating hydroxyapatite stabilization and reducing proteolytic collagen degradation [4,7]. P11-4 (Curodont™ Protect) forms a three-dimensional scaffold that mimics natural dentin matrix proteins, promoting mineral nucleation and growth [3,8]. SDF (Riva Star™) arrests dentinal caries by precipitating silver- and fluoride-containing compounds, enhancing mechanical strength, and inhibiting MMPs, thereby improving bonding substrates [6,9,10].

However, despite increasing evidence on the remineralizing potential of these agents, there is a lack of conclusive data regarding their effect on the bonding performance of universal adhesives when applied to caries-affected dentin. Moreover, the interaction between remineralization treatments and different adhesion strategies, etch-and-rinse versus self-etch, remains insufficiently explored.

Therefore, the aim of this study was to evaluate the influence of three remineralization treatments (CPP-ACP, P11-4, and SDF) on the microtensile bond strength of universal adhesives to caries-affected dentin. Additionally, the study compared the performance of two adhesive strategies to determine whether remineralization could enhance bonding effectiveness. The null hypothesis stated that neither remineralizing agents nor adhesive strategies would significantly affect bond strength.

## 2. Materials and Methods

The study was conducted following the approval of the Research Ethics Commission of the ‘Grigore T. Popa’ University of Medicine and Pharmacy in Iași (approval no: 291/10.04.2023), in full compliance with the principles outlined in the Declaration of Helsinki.

The sample size was calculated using G*Power software (version 3.1.9.7, Heinrich-Heine University, Düsseldorf, Germany), applying a medium effect size of 0.35, an alpha level of 0.05, and a statistical power of 0.80, which resulted in a minimum required total of 140 specimens. These parameters were selected based on previous research in adhesive dentistry [11,12], ensuring sufficient sensitivity to detect clinically relevant differences between treatment groups.

This in vitro study included 70 sound, caries-free human molars extracted for orthodontic or periodontal reasons. After extraction, the teeth were cleaned in an ultrasonic bath (Evo Sonic, Mettmann, Germany).

### 2.1. Preparation of Dentin Surfaces

**Figure 1:**
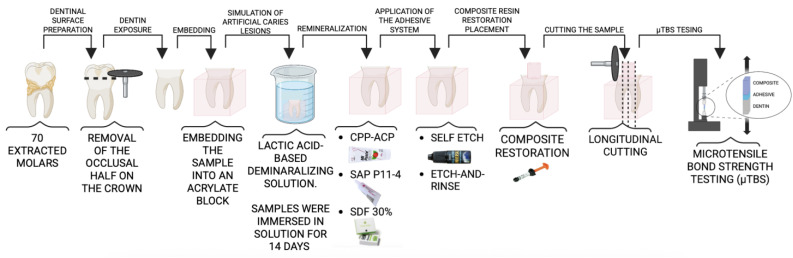
Study protocol.

The crowns were sectioned horizontally at mid-height, perpendicular to the long axis of the tooth, using a diamond disk mounted on a straight handpiece under water irrigation to expose the dentin. The exposed dentin surfaces were then gently polished with #600-grit sandpaper under continuous water cooling. Following this, the tooth roots were embedded in acrylic resin blocks (Duracryl PLUS, Spofa Dental, Jičín, Czechia) up to the level of the crown margin, leaving the flat dentin surface exposed. The complete experimental protocol is illustrated in Figure 1.

### 2.2. Induction of Artificial Carious Lesions on Dentin

All study samples, except for the control groups (C1 and C2), underwent a controlled demineralization process to simulate caries-affected dentin. The specimens were immersed in a lactic acid-based demineralization solution (0.1 M, pH 5.0) containing 1.5 mM/L CaCl_2_, 0.9 mM/L KH_2_PO_4_, 0.5 ppm fluoride (added via a standardized fluoride solution), and 0.1% chloramine T to ensure antimicrobial protection [2,3]. The solution volume (approximately 5 L) was sufficient to fully submerge all samples. After 7 days, the solution was replaced with a fresh batch, and the demineralization process continued for a total of 14 days at room temperature. This protocol is designed to produce artificial dentin lesions with mineral loss comparable to naturally caries-affected dentin, while preserving the integrity of the collagen matrix.

### 2.3. Remineralization Procedure

After induction of the artificial carious lesions, the demineralized dentin samples were distributed into 6 study groups (S1–S6; which received remineralization treatment) and 2 control groups (C3, C4; which did not receive remineralization treatment) as shown in Table 1.

The following strategies were used for the remineralization procedure. The composition of the remineralizing products and of the adhesive systems is presented in Table 2.

1. CPP-ACP Treatment

A paste containing casein phosphopeptide and amorphous calcium phosphate (CPP-ACP; MI Paste™, GC America, Alsip, IL, USA) was applied to the demineralized dentin surface using a cotton applicator for 3 min, once daily, for a period of 7 days. After each application, the surface was gently rinsed, and the samples were stored in artificial saliva at 37 °C in an incubator (Biobase BJPXH30II, Biodusty, Shandong, China), until the next treatment. A total of seven application cycles were completed. Following the final application, the samples remained stored in artificial saliva until the adhesive procedures were performed.

2. Self-Assembling Peptide P11-4 Treatment

The self-assembling peptide gel P11-4 (Curodont™ Protect, Credentis AG, Zug, Switzerland) was applied to the demineralized dentin in accordance with the manufacturer’s instructions. The gel was left undisturbed for approximately 5 min, allowing full absorption and penetration into the dentin matrix, evidenced by the visible disappearance of the gel. Then, the samples were gently rinsed and stored in artificial saliva at 37 °C for 7 days, with the solution replaced daily. This storage period allowed the biomimetic remineralization process to occur within the peptide-infiltrated collagen network.

3. Silver Diamine Fluoride (SDF) Treatment

A uniform layer of 38% silver diamine fluoride solution (Riva Star™, SDI Ltd, Bayswater, Victoria, Australia) was applied to the demineralized dentin surface and left to act for approximately 1 min. This was followed by the application of potassium iodide (KI), also provided by the manufacturer, until the initially formed creamy-white silver iodide (AgI) precipitate became clear, indicating neutralization of excess silver ions. This step was intended to minimize the dark staining typically associated with SDF treatment. The specimens were then stored in artificial saliva at 37 °C for 7 days, with daily solution changes, to facilitate mineral (such as calcium fluoride) deposition within the dentin.

4. Artificial Saliva Composition

The artificial saliva used for sample storage post-treatment was freshly prepared each day, based on a standard mucin-free formulation. It contained 0.65 g/L calcium chloride, 0.58 g/L dipotassium phosphate, 0.17 g/L KH_2_PO_4_, 1.27 g/L potassium chloride, 0.85 g/L sodium chloride, and 0.15 g/L potassium thiocyanate, with the pH adjusted to approximately 7.0 [13].

### 2.4. Application of Universal Bonding Systems and Composite Restoration

1. *Adhesive Procedure*

At the end of the remineralization protocol, or immediately after demineralization for control groups that did not receive remineralizing treatment, the dentin surfaces were thoroughly rinsed with distilled water and gently air-dried, ensuring the surface remained slightly moist to avoid collagen fiber collapse due to over-drying.

Two adhesive strategies were employed in this study:

2. *Etch-and-Rinse Strategy*

For the etch-and-rinse approach, the universal adhesive All-Bond Universal^®^ (BISCO, Schaumburg, IL, USA) was used, following the manufacturer’s instructions. First, the dentin surface was etched with 35% phosphoric acid gel for 15 s and then thoroughly rinsed with water for 10 s. The surface was gently blotted to remove excess moisture, taking care to keep it slightly moist so that the collagen network remained intact. Then, two layers of the adhesive were applied using a microbrush, with light rubbing for about 15 s to ensure good coverage. The solvent was gently evaporated using a stream of air for 5 s. The adhesive was then light-cured for 10 s with a LED curing lamp (Woodpecker LED.E, Giulin, Guangxi, China), operating at an intensity of 1000 mW/cm^2^ and a wavelength range of 420–480 nm.

3. *Self-Etch Strategy*

The universal adhesive All-Bond Universal^®^ was also employed in self-etch mode. This single-component adhesive was applied using a microbrush and actively rubbed onto the dentin surface (left unetched) for approximately 20 s. This procedure was performed in two consecutive applications to ensure thorough infiltration of the substrate. Following application, the adhesive was gently air-dried for five seconds to facilitate solvent evaporation, then polymerized for ten seconds using an LED curing unit, in accordance with the manufacturer’s protocol.

Then, after the adhesive was light-cured, all samples were restored using a nanohybrid composite resin (Filtek™ Z550, shade A2; 3M ESPE). The composite was applied in two increments to form a structure of approximately 3–4 mm above the dentin surface. Each increment, measuring roughly 1.5–2 mm in thickness, was polymerized for 20 s using the same LED curing unit, resulting in a composite build-up securely bonded to the underlying dentin. Once the composite application was complete, the specimens were immersed in distilled water and stored at 37 °C for a 24-h period prior to undergoing mechanical evaluation.

### 2.5. Determination of Microtensile Strength (μTBS)

To evaluate microtensile bond strength (μTBS), acrylate blocks containing the restored dental specimens were sectioned longitudinally using a Struers Secotom 50 precision cutting machine (Struers LLC, Cleveland, OH, USA) equipped with a water-cooled diamond blade measuring 0.5 mm in thickness. This procedure yielded between six and eight rod-shaped specimens per block, each approximately 2.5 cm in height and 1 mm in both width and depth. Due to the reduced dimensions of the resulting samples, the three most intact and structurally sound sticks were identified and selected from each set for subsequent microhardness assessment using a Lloyd LS5 universal testing machine (Ametek, Leicester, UK).

Each stick was fixed to the testing device using cyanoacrylate adhesive (Loctite 415), applied to both ends to ensure secure mounting. Tensile strength was assessed using a 100 N load cell, with force applied at a crosshead displacement rate of 1.0 mm/min until structural failure occurred. The recorded rupture forces (in Newtons) were used to compute the microtensile bond strength by dividing the force value by the cross-sectional area (1 mm^2^), with the final results expressed in megapascals (MPa). Additionally, the failure mode of each specimen was documented post-testing, categorized as adhesive failure at the dentin-composite interface, cohesive failure within either the dentin or composite, or a combination of both (mixed failure).

### 2.6. Statistical Analysis

Statistical analyses were performed using IBM SPSS Statistics software (version 29.0.0). The assumption of normality was evaluated using Shapiro–Wilk test, while homogeneity of variances was assessed with Levene’s test. For comparisons between two independent groups, the independent samples *t*-test was employed. In cases involving more than two groups, one-way analysis of variance (ANOVA) was conducted, followed by Tukey’s post hoc test for pairwise comparisons. A threshold of *p* < 0.05 was used to determine statistical significance.

## 3. Results

The assessment of microtensile bond strength (μTBS) demonstrated statistically significant differences among the experimental groups, primarily influenced by dentin condition (sound vs. caries-affected), the type of remineralizing treatment applied, and the adhesive strategy employed.

Control groups involving sound dentin, C1: etch-and-rinse and C2: self-etch, exhibited the highest μTBS values, with mean strengths of 41.3 ± 2.7 MPa and 39.9 ± 3.8 MPa, respectively. These values serve as benchmarks for evaluating bonding performance on structurally compromised substrates.

**Figure 2:**
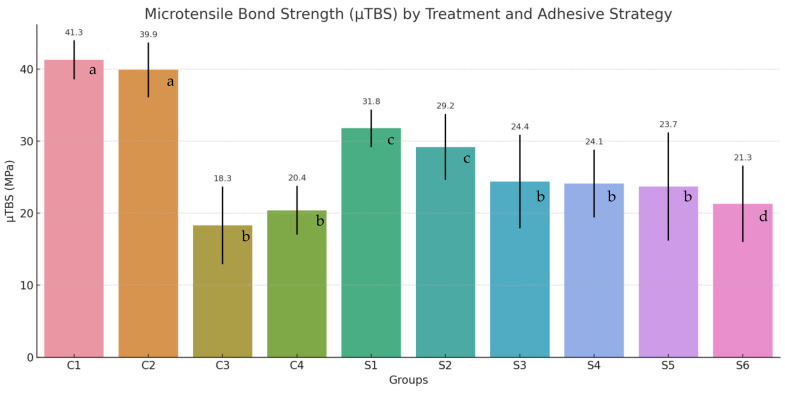
Mean microtensile bond strength (μTBS) values and standard deviations (error bars) for each experimental group. Groups C1 and C2 represent sound dentin (etch-and-rinse and self-etch strategies, respectively). Groups C3 and C4 correspond to untreated caries-affected dentin. Groups S1–S6 include caries-affected dentin treated with CPP-ACP (S1, S2), self-assembling peptide P11-4 (S3, S4), or silver diamine fluoride (S5, S6), followed by either etch-and-rinse (odd-numbered) or self-etch (even-numbered) universal adhesive application. CPP-ACP yielded the highest bond strength among remineralization strategies. Groups sharing the same superscript letter are not significantly different according to Tukey’s HSD test (*p* ≥ 0.05). Different superscript letters indicate significant differences between groups (*p* < 0.05).

In the absence of remineralization, caries-affected dentin showed markedly reduced μTBS values: 18.3 ± 5.4 MPa for group C3 (etch-and-rinse) and 20.4 ± 3.4 MPa for group C4 (self-etch), highlighting the adverse impact of demineralization on adhesive integrity (Figure 2).

The use of remineralization protocols led to a statistically significant enhancement in microtensile bond strength (μTBS) across all evaluated conditions.

Among the remineralizing agents, casein phosphopeptide-amorphous calcium phosphate (CPP-ACP) demonstrated the most favorable outcomes, yielding μTBS values of 31.8 ± 2.6 MPa for the etch-and-rinse protocol (Group S1) and 29.2 ± 4.6 MPa for the self-etch approach (Group S2). Those values were closest to those recorded for sound dentine (C1: 41.3 ± 2.7 MPa; C2: 39.9 ± 3.8 MPa), indicating a high degree of functional recovery.

The self-assembling peptide P11-4 exhibited moderate efficacy, with μTBS values of 24.4 ± 6.5 MPa for etch-and-rinse (Group S3) and 24.1 ± 4.7 MPa for self-etch (Group S4), showing minimal discrepancy between adhesive strategies.

Silver diamine fluoride (SDF), produced more modest gains in bond strength. The recorded μTBS values were 23.7 ± 7.5 MPa for etch-and-rinse strategy (Group S5) and 21.3 ± 5.3 MPa for self-etch (Group S6), which, while higher than those observed in untreated caries-affected dentin (Group C3 with 18.3 ± 5.4 Mpa and C4 with 20.4 ± 3.4 MPa), remained lower than the outcomes achieved with CPP-ACP and P11-4.

Statistical validation using analysis of variance (ANOVA) and Tukey’s post hoc test confirmed the presence of significant differences (*p* < 0.05) both between remineralized and non-remineralized groups, as well as among the individual treatment modalities. Even if the differences were not statistically significant, the etch-and-rinse adhesive protocol consistently outperformed the self-etch approach in most scenarios, particularly in samples treated with P11-4 or SDF, as well as in untreated dentin.

Microtensile bond strength (μTBS) values (mean ± SD, MPa) for each group, and *p*-values from independent samples *t*-tests comparing etch-and-rinse vs. self-etch within each treatment type are presented in Table 3.

Before conducting inferential statistical analyses, the assumptions of data normality and homogeneity of variances were assessed using the Shapiro–Wilk and Levene’s tests, respectively. All datasets met these assumptions (*p* > 0.05), thereby justifying the application of parametric methods.

To examine the effect of adhesive strategy, etch-and-rinse versus self-etch, within each remineralization treatment group, independent samples *t*-tests were performed. The analyses revealed no statistically significant differences in bond strength between the two adhesive approaches across any of the experimental conditions, including the control groups. These findings suggest that, when applied to the same dentin substrate, the mode of universal adhesive application does not exert a significant influence on bonding performance.

To evaluate differences among the various treatment conditions, separate one-way analyses of variance (ANOVA) were conducted for the etch-and-rinse (ER) and self-etch (SE) protocols, followed by Tukey’s HSD post hoc tests. Both analyses revealed statistically significant differences across the five treatment groups within each adhesive strategy (ER: F = 19.49, *p* < 0.001; SE: F = 22.70, *p* < 0.001).

Within the etch-and-rinse group, specimens in the sound dentin control (C1) demonstrated significantly higher microtensile bond strength (μTBS) values compared to all other ER groups (*p* < 0.001). Among the remineralized samples, the CPP-ACP-treated group (S1) achieved significantly greater bond strength than the demineralized control (C3) (*p* < 0.001), and also outperformed the P11-4 (S3) and SDF (S5) groups, although not all comparisons reached statistical significance.

Comparable trends were observed in the self-etch protocol. The sound dentin control (C2) exhibited significantly higher μTBS values than all other SE groups (*p* < 0.001). CPP-ACP application (S2) resulted in significantly enhanced bond strength relative to both the untreated caries-affected dentin (C4, *p* = 0.007) and the SDF-treated group (S6, *p* = 0.018). In contrast, the group treated with P11-4 (S4) did not demonstrate statistically significant improvements compared to C4 or S6.

## 4. Discussion

The findings of this in vitro investigation highlight the potential benefits of applying remineralizing agents to enhance the bonding performance of universal adhesive systems on caries-affected dentin. As anticipated, demineralized and untreated dentin exhibited a marked reduction in bond strength which is an outcome that aligns with previously published research [10,14]. Meta-analyses have consistently demonstrated that adhesive systems achieve significantly lower bond strengths on carious dentin compared to sound dentin, with reported differences typically ranging from 6 to 10 MPa, depending on the used adhesive generation [14].

This decrease in bonding efficacy can be attributed to the altered structural and biochemical characteristics of demineralized dentin. Specifically, this type of dentin often presents areas of exposed collagen that are inadequately infiltrated by adhesive resins, as well as water retention at the bonding interface. These factors contribute to the formation of a hybrid layer of suboptimal quality, prone to degradation over time [15,16,17,18]. Moreover, the activation of endogenous proteolytic enzymes, particularly matrix metalloproteinases (MMPs) and cathepsins, within demineralized dentin further compromises the longevity of the adhesive interface by promoting post-polymerization degradation [19].

In clinical scenarios that emphasize minimally invasive treatment strategies, where partially demineralized dentin is intentionally preserved, it becomes increasingly important to explore methods for reinforcing this compromised substrate prior to adhesive application. Within this context, the use of remineralizing agents emerges as a promising approach, as supported by the outcomes observed in the present study.

The findings of the present study indicate that the pre-treatment of caries-affected dentin with remineralizing agents represents a promising approach to enhance the quality of the substrate and, by extension, improve adhesive performance. Among the tested agents, casein phosphopeptide-amorphous calcium phosphate (CPP-ACP) demonstrated particularly favorable outcomes. Its efficacy appears to stem from its capacity to deliver bioavailable calcium and phosphate ions into the interfibrillar spaces of demineralized collagen matrices. This ion exchange facilitates partial remineralization through the reformation of apatite crystals, thereby reinforcing the mechanical integrity of the dentin structure [20].

Importantly, this pre-treatment modifies the dentinal response to subsequent acid etching. When phosphoric acid is applied to dentin previously conditioned with CPP-ACP, the extent of demineralization is significantly reduced [21]. The collagen network remains more stably supported by mineral content, thereby preventing collapse and preserving the structural scaffold necessary for effective adhesive infiltration [20]. As a result, the formation of the hybrid layer is more consistent and mechanically resilient, contributing to improved bond strength [21].

The results of a previous study supports the use of CPP-ACP in enhancing adhesion to compromised dentin [20]. Multiple studies have reported significant increases in bond strength when CPP-ACP is used as a supplementary conditioning agent [22]. One critical practical consideration concerns the duration of application. Prior research has indicated that a minimum application time of approximately three minutes is necessary to elicit a measurable effect, whereas shorter exposure times ranging from 30 to 90 s tend to be insufficient [21]. In the present study, CPP-ACP was applied daily in repeated three-minute sessions, a regimen that appears to ensure adequate mineral infiltration of the dentin substrate. Clinically, this approach can be translated into practice by applying a CPP-ACP–based formulation (such as MI Paste) directly into the prepared cavity for several minutes, either during multiple appointments, in a single dedicated session prior to final restoration placement, or as part of a structured home-care protocol. Repeated short-duration applications enhance mineral deposition and improve the structural integrity of the dentin substrate, thereby creating more favorable conditions for adhesive procedures, particularly in deep carious lesions.

Peptide P11-4 represents an emerging biomimetic strategy, originally introduced for the remineralization of early enamel lesions [23]. In the present study, its application has been expanded to caries-affected dentin. P11-4 exhibits the capacity to self-assemble into nanofibrous structures within the moist microenvironment of the lesion, where it acts as a scaffold for the nucleation and growth of apatite crystals [24]. In the context of demineralized dentin, these peptides are capable of diffusing into the porous collagen framework. Once in place, they facilitate the in situ formation of mineral deposits along their structure, which can significantly reinforce the compromised dentin [15]. Sakr et al. provided ultrastructural evidence through electron microscopy, demonstrating that specimens treated with P11-4 displayed a reduction in nanoporosity within the hybrid layer and decreased nanoleakage following accelerated aging protocols, in comparison to untreated controls. These findings suggest an enhanced long-term stability of the adhesive interface in P11-4 pre-treated dentin [23].

An important consideration emerging from the literature is the interaction between P11-4 and the type of adhesive system employed. Barbosa-Martins et al. reported a significant improvement in microtensile bond strength (μTBS) when P11-4 was used in conjunction with an etch-and-rinse adhesive. However, no comparable benefit was observed when a mild self-etch adhesive, such as Clearfil SE Bond, was applied [5]. This differential effect is likely attributable to the limited etching capacity of such adhesives (pH ~2), which may be insufficient to dissolve the P11-4-induced mineral layer. As a result, a residual mineralized surface may act as a diffusion barrier, impeding adequate resin infiltration and hybrid layer formation [24,25]. Conversely, the phosphoric acid used in total-etch protocols can effectively remove this superficial layer, exposing the underlying remineralized dentin and facilitating better adhesive integration [26,27].

Clinically, these findings underscore the importance of adhesive strategy selection when incorporating P11-4 into restorative protocols. Specifically, the use of total-etch or selective-etch techniques may be more compatible with P11-4 pretreatment, maximizing the benefits of localized remineralization while preserving the integrity of the adhesive interface [27]. Unlike CPP-ACP, which requires repeated applications to achieve clinical effectiveness, P11-4 offers the advantage of a single application. Moreover, its potential to maintain long-term adhesive interface integrity makes it an attractive option in restorative dentistry [28]. Nonetheless, limitations related to cost and accessibility must be acknowledged, as P11-4 remains a relatively novel product with limited availability in routine clinical practice.

Silver diamine fluoride (SDF) is a well-established agent for arresting caries, particularly in pediatric patients and root lesions, due to its strong antimicrobial activity and dentin-strengthening properties. However, its influence on composite resin adhesion has generated debate. Some studies have reported that when SDF is left unaltered on the dentin surface, it may impair bonding, likely due to the formation of insoluble precipitates (such as AgCl, Ag_3_PO_4_) that occlude tubules and hinder proper resin infiltration [24]. Conversely, research has shown that rinsing, wiping, or lightly abrading the surface after SDF application can mitigate this negative effect, resulting in bond strength comparable to untreated dentin [29,30].

In our study, the protocol involved rinsing the surface and storing samples in artificial saliva, likely reducing residual reactive silver. Under such conditions, a slight increase in microtensile bond strength (μTBS) was observed, especially when using total-etch adhesive, but with no statistical significance. This result aligns with previous findings suggesting that SDF may enhance bonding to caries-affected dentin [29]. Potential mechanisms include the deposition of calcium fluoride and silver phosphates within the collagen matrix, contributing to increased hardness, alongside inhibition of matrix metalloproteinases (MMPs), protection against collagen degradation, and elimination of residual bacteria [30,31].

Nonetheless, the durability of this benefit remains uncertain. Some studies have shown that after six months of aging, the initial increase in μTBS was no longer evident, likely due to leaching or displacement of silver and CaF_2_ compounds over time [32]. If the collagen network has not been adequately remineralized at the intrafibrillar level, it may remain vulnerable to hydrolytic degradation once these protective compounds are lost [30,32].

Thus, while SDF offers an appealing, clinically simple approach for dentin reinforcement, adjunctive strategies may be necessary to optimize bonding outcomes. These include surface polishing after SDF application, use of a hydrophilic primer to enhance resin infiltration, or the selection of bioactive ionomer-based materials, which may adhere more predictably to SDF-treated dentin [21]. When using composite resins, the simultaneous application of potassium iodide, as performed in our protocol, can help reduce staining and excess silver. This should be followed by thorough rinsing and, if feasible, mechanical conditioning (e.g., brushing or air-abrasion) prior to adhesive placement [31,33].

An important consideration is the choice of adhesive strategy for remineralized carious dentin. Our findings indicate that universal adhesives applied in the etch-and-rinse mode generally yielded equal or superior bond strength compared to the self-etch mode, aligning with previous evidence from Isolan et al. [14]. This can be attributed to phosphoric acid’s ability to effectively remove the smear layer and expose intact collagen fibrils, enabling better primer penetration, even in porous, affected dentin [34].

In contrast, self-etch systems may inadequately condition caries-affected dentin, resulting in weaker adhesion. However, when dentin was pre-treated with remineralizing agents, like CPP-ACP, the differences between etching modes diminished. This suggests that substrate remineralization enhances surface energy and wettability, facilitating adhesive infiltration regardless of the etching technique [35]. Interestingly, some studies have shown that, following minimally invasive caries removal, self-etch adhesives can achieve comparable or even improved performance over etch-and-rinse strategies [10,35]. These variations highlight that optimal adhesion depends strongly on substrate condition. Although etch-and-rinse application may promote a thicker hybrid layer and slightly better bond strength [14,21,25], it may also expose collagen, necessitating MMP inhibition. On the other hand, self-etch adhesives, though milder, may benefit from the mechanical reinforcement provided by remineralization.

Our findings are consistent with prior research confirming the efficacy of CPP-ACP in enhancing adhesion to demineralized dentin [20]. Additionally, various studies support the benefits of self-assembling peptides like P11-4 and collagen-stabilizing agents (e.g., glutaraldehyde, CHX) in preserving adhesive interface integrity [18,21]. In the case of silver diamine fluoride (SDF), literature shows mixed results. A systematic review by Jiang et al. found that while many studies reported no adverse effect, especially when SDF was rinsed, others observed reduced bond strength [29]. These discrepancies likely reflect differences in SDF protocols, including concentration, dwell time, rinsing, and adhesive type.

Emerging guidelines recommend thorough rinsing or wiping of SDF and superficial abrasion of the treated dentin to remove the silver phosphate-rich layer and expose a cleaner, bioactive surface enriched with CaF_2_ and collagen-bound silver [18]. Using an adhesive with strong penetration capacity (e.g., universal systems applied in double layers) may further improve bonding. In our study, the use of potassium iodide (KI) prevented staining, suggesting that clinical application of commercial systems like Riva Star (SDF + KI) could allow immediate esthetic restorations. However, when plain SDF is used, removing the superficial dentin layer (approx. 0.1–0.2 mm) before bonding is advisable to prevent adhesion issues [10].

This study presents several limitations that should be considered when interpreting the findings. Firstly, its in vitro design limits the direct extrapolation of the results to clinical conditions, as key factors such as long-term hydrolytic degradation, thermocycling, and mechanical fatigue were not assessed. Furthermore, no artificial aging protocols, including thermocycling or extended water storage, were employed, which could have influenced the durability and stability of the adhesive interface. The conclusions are also restricted to artificially demineralized dentin, which may not accurately reflect the behavior of naturally carious dentin, highlighting the need for additional in vivo studies to confirm clinical relevance. The long-term effectiveness of the tested agents also remains uncertain: while P11-4 may support sustained stability, CHX offers only short-term MMP inhibition, and the effect of SDF may gradually diminish. Moreover, this study did not investigate the potential staining associated with remineralizing agents, an aspect of clinical importance, particularly in visible areas where CPP-ACP and P11-4 could represent more suitable alternatives to SDF. Lastly, the lack of a failure mode analysis restricts a deeper understanding of the adhesive interface performance, indicating an important direction for future research.

The findings support remineralization of caries-affected dentin as a promising strategy to enhance adhesive performance, complementing established methods such as collagen cross-linkers (e.g., proanthocyanidins) and MMP inhibitors like chlorhexidine. While the latter chemically stabilize collagen without restoring mineral content, agents like CPP-ACP, P11-4, and SDF promote dentin remineralization and modulate its organic matrix. This bioactive approach aligns with current minimally invasive principles, aiming to conserve and restore natural tooth structure before restoration.

However, the clinical relevance of these benefits remains to be fully established. Future controlled trials are needed to determine whether dentin pre-treatment with materials such as MI Paste or self-assembling peptides can reduce restorative failures (like marginal degradation or secondary caries) in deep cavities compared to conventional techniques.

## 5. Conclusions

- All three treatments (CPP-ACP, P11-4, SDF) enhanced the microtensile bond strength (μTBS) of universal adhesives to caries-affected dentin compared to untreated dentin.
- CPP-ACP (MI Paste) showed the highest μTBS values among all treatments, approaching those of sound dentin, in both etch-and-rinse and self-etch modes.
- The self-assembling peptide P11-4 led to moderate improvements in bond strength, with minimal differences between the two adhesive strategies.
- Silver diamine fluoride (SDF), applied with potassium iodide, modestly increased bond strength but was less effective than CPP-ACP and P11-4.
- While not statistically significant, the etch-and-rinse strategy generally yielded higher μTBS values than the self-etch approach, especially in untreated or less effectively remineralized dentin.

## Figures and Tables

**Table 1:** The distribution of the samples in groups.

Group	Demineralization	Universal Adhesive System Application in Etch and Rinse Strategy	Universal Adhesive System Application in Self-Etch Strategies	Remineralization with CPP-ACP Product	Remineralization with Self-Assembling Peptides P11-4	Remineralization with SDF	Composite Resin
C1 (Control 1)		X					X
C2 (Control 2)			X				X
C3 (Control 3)	X	X					X
C4 (Control 4)	X		X				X
S1 (Study 1)	X	X		X			X
S2 (Study 2)	X		X	X			X
S3 (Study 3)	X	X			X		X
S4 (Study 4)	X		X		X		X
S5 (Study 5)	X	X				X	X
S6 (Study 6)	X		X			X	X

**Table 2:** The composition of the remineralizing products and adhesive systems.

Material	Composition
MI Paste™ (CPP-ACP)	CPP-ACP (casein phosphopeptide-amorphous calcium phosphate), water, glycerin, sorbitol
Curodont™ Protect (Self-Assembling Peptide P11-4)	Self-assembling peptide (SAP P11-4), sodium fluoride (1450 ppm F^−^), xylitol
SDF Riva Star™ (Silver Diamine Fluoride)	Solution 1: 38% silver diamine fluoride (SDF); Solution 2: potassium iodide (KI)
All-Bond Universal	MDP (10-Methacryloyloxydecyl dihydrogen phosphate), Bis-GMA, HEMA, solvent (ethanol), water, photoinitiator

**Table 3:** Microtensile bond strength (μTBS) values (mean ± SD, MPa) for each group, and *p*-values from independent samples *t*-tests comparing etch-and-rinse vs. self-etch within each treatment type.

Group	μTBS ± SD (MPa)	*p* Value
C1	41.3 ± 2.7	0.218
C2	39.9 ± 3.8	
C3	18.3 ± 5.4	0.406
C4	20.4 ± 3.4	
S1	31.8 ± 2.6	0.224
S2	29.2 ± 4.6	
S3	24.4 ± 6.5	0.923
S4	24.1 ± 4.7	
S5	23.7 ± 7.5	0.504
S6	21.3 ± 5.3	

## Data Availability

The original contributions presented in this study are included in the article. Further inquiries can be directed to the corresponding author.

## Abbreviations

The following abbreviations are used in this manuscript:

μTBS	Microtensile Bond Strength
CPP-ACP	Casein Phosphopeptide-Amorphous Calcium Phosphate
P11-4	Self-Assembling Peptide P11-4
SDF	Silver Diamine Fluoride
KI	Potassium Iodide
MMPs	Matrix Metalloproteinases
HEMA	2-Hydroxyethyl Methacrylate
Bis-GMA	Bisphenol A-Glycidyl Methacrylate
MDP	10-Methacryloyloxydecyl Dihydrogen Phosphate
LED	Light-Emitting Diode
SD	Standard Deviation
ER	Etch-and-Rinse
SE	Self-Etch
CHX	Chlorhexidine
AgI	Silver Iodide
CaF_2_	Calcium Fluoride
Ag_3_PO_4_	Silver Phosphate
